# Correction: Risk Factors for Sporadic Non-Pregnancy Associated Listeriosis in Germany—Immunocompromised Patients and Frequently Consumed Ready-To-Eat Products

**DOI:** 10.1371/journal.pone.0165990

**Published:** 2016-10-27

**Authors:** The PLOS ONE Staff

## Notice of Republication

This article was republished on January 8, 2016, to correct an error in the legend of Fig 1 that was introduced during the typesetting process. The publisher apologizes for the error. Please download this article again to view the correct version. The originally published, uncorrected article and the republished, corrected article are provided here for reference.

## Supporting Information

S1 File Originally published, uncorrected article. (PDF)

S2 File Republished, corrected article. (PDF)
