# Supplementary material for: Correction: Risk Factors for Sporadic Non-Pregnancy Associated Listeriosis in Germany—Immunocompromised Patients and Frequently Consumed Ready-To-Eat Products
Source: PLoS One. 2016 Oct 27;11(10):e0165990. doi: 10.1371/journal.pone.0165990 (PMC5082834; doi:10.1371/journal.pone.0165990)
Supplement: S1 File — (PDF) [file pone.0165990.s001.pdf]

PLOS | ONE

RESEARCH ARTICLE

Risk Factors for Sporadic Non-Pregnancy Associated Listeriosis in Germany—Immunocompromised Patients and Frequently Consumed Ready-To-Eat Products

Karina Preußel[1]*, Astrid Milde-Busch[1], Patrick Schmich[2], Matthias Wetzstein[2], Klaus Stark[1], Dirk Werber[1¤]

1 Department for Infectious Disease Epidemiology, Robert Koch Institute, Berlin, Germany, **2** Department of Epidemiology and Health Monitoring, Robert Koch Institute, Berlin, Germany

¤ Current address: Berlin Unit for Infectious Disease Surveillance and Environmental Health, State Office for Health and Social Affairs, Berlin, Germany
* preusselk@rki.de

CrossMark
click for updates

OPEN ACCESS

Citation: Preußel K, Milde-Busch A, Schmich P, Wetzstein M, Stark K, Werber D (2015) Risk Factors for Sporadic Non-Pregnancy Associated Listeriosis in Germany—Immunocompromised Patients and Frequently Consumed Ready-To-Eat Products. PLoS ONE 10(11): e0142986. doi:10.1371/journal.pone.0142986

Editor: Martyn Kirk, The Australian National University, AUSTRALIA

Received: June 8, 2015

Accepted: October 29, 2015

Published: November 23, 2015

Copyright: © 2015 Preußel et al. This is an open access article distributed under the terms of the Creative Commons Attribution License, which permits unrestricted use, distribution, and reproduction in any medium, provided the original author and source are credited.

Data Availability Statement: The data set underlying the findings is available from the Zenodo repository (https://zenodo.org/record/32864; DOI 10.5281/zenodo.32864). Patient privacy concerns make raw study data legally and ethically unsuitable for public posting. To ensure anonymity of the patients, age classes and underlying conditions were provided as composite variables.

Funding: The authors have no support or funding to report.

Abstract

Non-pregnancy associated (N-PA) listeriosis, caused by *Listeria monocytogenes*, is a rare but severe disease, and is predominantly food-borne. Most cases appear sporadic and their infection vehicle remains unknown. Incidence has increased since 2008 in Germany. We aimed to identify underlying conditions and foods associated with sporadic N-PA listeriosis in Germany. We performed a nationwide case-control study from March 2012-December 2013. Cases were sporadic N-PA listeriosis patients notified to public health. Control subjects were age (40–65 years, 66–75 years, $\geq$76 years) frequency-matched persons from a nationwide random telephone sample. A structured questionnaire collected information on underlying diseases, therapies and >60 food items. We conducted multivariable logistic regression analysis, adjusting for host factors identified by causal diagram theory, and calculated population attributable fractions. We enrolled 109 cases and 1982 controls. Cases' median age was 69 years, 55% were male, 44% received immunosuppressive therapy within 3 months prior to illness onset; a further 28% had at least one immunocompromising disease. In multivariable analysis, immunosuppressive therapy (OR 8.8, 95%CI 4.9–15.6), immunocompromising disease (OR 2.7; 95% CI 1.4–5.2), gastric acid suppression (OR 3.0; 95%CI 1.4–6.3), the consumption of cold cooked sausages (OR 2.6; 95%CI 1.6–4.4), the preferred consumption of packaged cheese (OR 2.1; 95%CI 1.3–3.5) and pre-sliced cheese (OR 2.2; 95%CI 1.3–3.7) were significantly associated with N-PA listeriosis. These foods accounted for 59% of all cases. Typical high risk foods, e.g. cold seafood, certain types of cheeses, tended to be negatively associated with disease. In conclusion, immunosuppressive therapy and frequently consumed ready-to-eat foods are the main risk factors for sporadic N-PA listeriosis in Germany. To reduce their risk, immunocompromised persons should consume the identified foods well before the 'use-by' date. The microbiological criteria for *Listeria monocytogenes* in ready-to-eat foods may insufficiently protect persons who are markedly immunocompromised.

Competing Interests: The authors have declared that no competing interests exist.

Introduction

Listeriosis is a rare but life-threatening disease with high mortality, caused by the bacterium *Listeria monocytogenes*. Host factors relating to reduced immunocompetency are known to play an important role in listeriosis. Cases occur predominantly in the elderly and in immunocompromised persons (non-pregnancy associated, 'N-PA'), and in pregnant women. Moreover, gastric acid suppression increases susceptibility to bacterial enteric infection [1], but an association with listeriosis independent of underlying diseases has not been demonstrated thus far. In adults, common manifestations of listeriosis are meningoencephalitis, sepsis and focal infections in various organ systems.

L. monocytogenes is transmitted predominantly through contaminated food [2]. Various food vehicles have been identified in outbreaks [3–5] including ready-to-eat meat products (e.g. frankfurters), pasteurized and unpasteurized cheese (e.g. soft cheeses), fruit, vegetables and fish products. Case-control studies of risk factors for sporadic illness have produced highly inconsistent results. One failed to identify food items at all [6], others found statistical associations with different foods that could often explained only a minority of the cases [7–9], including hummus and ice milk [9], ready-to-eat beef, smoked fish, prawns, milk, butter, cheese and mixed salads [7, 10]. The importance of these foods or other risk factors for sporadic N-PA listeriosis in Germany has not been systematically investigated so far.

In Germany, detection of *L. monocytogenes* in cultures from a normally sterile site, usually from blood or cerebrospinal fluid, is notifiable to the local Public health department. Most notified cases appear sporadic, are N-PA (>90%), and the source of infection remains unknown. Listeriosis is associated with the highest mortality (7–9% of notified cases, 2009–2013) and years of potential life lost among classical notifiable enteric pathogens [11], even when neglecting the contribution of pregnancy associated cases. Annual incidence of notified N-PA listeriosis has increased from 0.3 per 100 000 population in 2008 to 0.5 per 100 000 population in 2013. We aimed to identify underlying conditions and foods associated with N-PA listeriosis.

Methods

Study Design and Data Collection

We conducted an age frequency-matched case-control study in all German Federal States, except for the Federal city state of Bremen (0.8% of the German population), and enrolled cases prospectively from 15 March 2012 (Baden-Württemberg: 29 May 2012) to 31 December 2013. A case was defined as clinically compatible illness (fever, septicaemia, meningitis or localized organ infection) in a non-pregnant person with isolation of *L. monocytogenes* from a normally sterile site who was notified to a local health department during the study period, but not part of a recognized outbreak for which a common vehicle of infection was identified.

Staff from local health authorities contacted case patients to request informed verbal consent and documented patients' response. Obtaining written informed consent was deemed to be associated with substantial time delay, thereby reducing patients' food history recall, particularly as most patients were still in hospital when contacted by telephone. Consent procedure was approved by the ethics committee.

Case interviews were conducted by staff from the local health authorities or the Robert Koch Institute (RKI). If a case patient was too ill or deceased, proxy interviews with persons familiar with the patient's food history or dietary habits were attempted.

Population-based control subjects were nationwide identified by random sampling of telephone numbers continuously between March 2012 and October 2013. The sample was

generated by the GESIS Leibniz Institute for Social Sciences, Mannheim according to the method by Gabler and Häder [12], taking into account telephone numbers not registered in telephone books. Computer-assisted telephone interviews (CATI) were conducted by the RKI targeting the oldest member of the household. To ensure balanced completion of control-strata, enrollment was split in blocks of 35 persons, i.e. recruitment was interrupted in a stratum after enrollment of 35 participants until the other strata had reached an equal number of enrolled control-subjects. Eligible control subjects had no known history of listeriosis and had to be accessible by telephone in Germany. Control subjects were frequency-matched by age-categories (40–65 years, 66–75 years, ≥76 years), which represented the terciles of the age distribution of reported N-PA-cases between 2004 and 2011. We attempted to recruit four controls per case (n = 400) to be able to identify associations with an odds ratio (OR) of 1.45 as statistically significant (1-β = 0.9; α = 0.05).

The structured questionnaire collected information on socio-demographic characteristics, symptoms of illness (only for cases), knowledge of the disease and transmission routes for listeriosis, underlying diseases, medication, and >60 items of food consumption and purchase during the 4-weeks prior to symptom onset (for cases) or prior to the interview (for controls). Information on underlying conditions was elicited for a) diseases accompanied with reduced immunocompetency and b) immunosuppressive therapies in the 3 months prior to illness onset (cases) or to interview (controls). For a list of conditions see Table 1.

Table 1. Association* of underlying conditions with non-pregnancy associated listeriosis in in a case control study in Germany, 2012–2013.

	Cases exposed		Controls exposed		OR*	95% CI	p-value
	n	%	n	%			
**Conditions associated with N-PA listeriosis**							
immunosuppressive therapy [a]							
Chemotherapy	14/108	13.0	17/1976	0.9	17.12	8.17–35.78	<0.001
Immunosupressive medication	40/98	40.8	189/1960	9.6	6.52	4.23–10.04	<0.001
Radiation therapy	5/108	4.6	17/1978	0.9	5.77	2.08–16.03	0.001
Hemodialysis	4/108	3.7	14/1901	0.7	5.44	1.75–16.96	0.003
immunocompromising disease							
Cancer–hemotological [b]	9/109	8.3	12/1978	0.6	15.97	6.51–39.16	<0.001
Splenectomy	5/106	4.7	9/1975	0.5	10.44	3.42–31.85	<0.001
Liver disease	13/106	12.3	40/1973	2.0	7.14	3.67–13.89	<0.001
Renal disease	17/108	15.7	86/1971	4.4	4.62	2.60–8.22	<0.001
Solid organ transplantation	4/107	3.7	22/1977	1.1	3.89	1.30–11.62	0.015
Autoimmune disorder	9/102	8.8	50/1934	2.6	3.53	1.68–7.41	0.001
Diabetes	29/107	27.1	306/1976	15.5	2.19	1.40–3.45	0.001
Non-immunological conditions							
Gastric acid suppression [a]	17/103	16.5	98/1963	5.0	3.84	2.19–6.72	<0.001
**Conditions without association to N-PA listeriosis**							
Hypertony	56/106	52.8	999/1972	50.7	1.18	0.79–1.77	0.426
Chronic enteritis	6/107	5.6	74/1965	3.8	1.52	0.65–3.58	0.338
Arthritis	18/99	18.2	289/1954	14.8	1.37	0.81–2.34	0.243
Cancer—solid (last 5 years)	16/106	15.1	208/1973	10.5	1.63	0.93–2.84	0.088
**No precondition**	11/78	14.1	592/1783	33.2	0.28	0.14–0.54	<0.001

* single variable analysis, adjusted for age

[a] within the last 3 months

[b] within the last 5 years

doi:10.1371/journal.pone.0142986.t001

Data from case patients was entered into an EpiData database (version 3.1, The EpiData Association, Denmark) and validated by double data entry. Data from controls were extracted from the CATI database (Voxco, Montreal, Canada) and merged with case data.

Data Analysis

Information for the person's highest educational background was classified as "high" (polytechnic or university degree), "medium" (apprenticeship or [advanced] vocational education), or "low" (no or current training) and used as an indicator for socioeconomic status (SES) [13]. Type of residence was classified as "village" (<2000 inhabitants), "town" (2000–100 000 inhabitants) or "metropolis" (>100 000 inhabitants), according to the definition of Germany's Federal Statistical Office. Food exposure data, which were collected in four frequency categories (from "never" to "daily") were dichotomized. Additionally, we combined various types of cheese that had a negative association with N-PA listeriosis ($p<0.05$) into a single variable that represented the consumption of at least one negatively associated type of cheese.

The case population was described with respect to frequencies of socio-demographic characteristics, underlying conditions and main disease outcomes.

Cases with information elicited from proxies were excluded from analysis of previous knowledge about listeriosis. Furthermore, knowledge about listeriosis and disease transmission in the study population was investigated with respect to underlying conditions.

Univariable analysis was performed for host variables and for all food related exposure variables. We computed univariable OR and 95% confidence intervals (95% CI) using logistic regression, adjusted for the matching factor age-category. Underlying conditions significantly associated with N-PA listeriosis and known to be associated with reduced immunocompetency [14–18] were grouped into "immunosuppressive therapy in the 3 months prior to illness onset (case) or interview (controls)", e.g. chemotherapy, and "immunocompromising diseases" without immunosuppressive therapy, e.g. diabetes. These categories were mutually exclusive and were compared against "no relevant condition", i.e. none of the conditions included in the former categories (see Table 1). Gastric acid suppression was considered as separate host factor, since we assumed that gastric acid suppression increases susceptibility to enteric infections primarily by non-immunologic mechanisms, thus distancing it from the mechanisms of immunocompromising diseases and therapies.

Multivariable logistic regression modeling was used with the primary objective of investigating the independent relationship of single food-exposures and N-PA listeriosis, while adjusting for the potential confounding effect of host factors. Thereafter, we applied a-two step strategy. First, we constructed directed acyclic graphs ('causal diagrams') to visualize the hypothesized causal structure of host factors and N-PA listeriosis. The relationships displayed in the graph were based on statistical associations identified in epidemiological, clinical and nutritional studies, official statistics, or, if the former was lacking, plausible assumptions (Fig 1). Because analytical adjustment for potential confounding variables can create bias where none exist [19], we deduced the minimally sufficient adjustment set to minimize bias using the graphical interface DAGitty [20]. Second, we then employed a manual forward stepwise selection strategy with the forced-in variables of the minimally sufficient adjustment set and food-items as candidate variables that had a p-value <0.05 in univariable analysis. Variables for points of food purchase and supply were not considered for multivariable modelling because of too many missing values. We set the p-value for candidate variables eligible for multivariable modeling narrowly to $p<0.05$ to reduce the risk of increasing bias and model non-convergence due to too few outcome events per explanatory variable [21]. Main-effects of the final

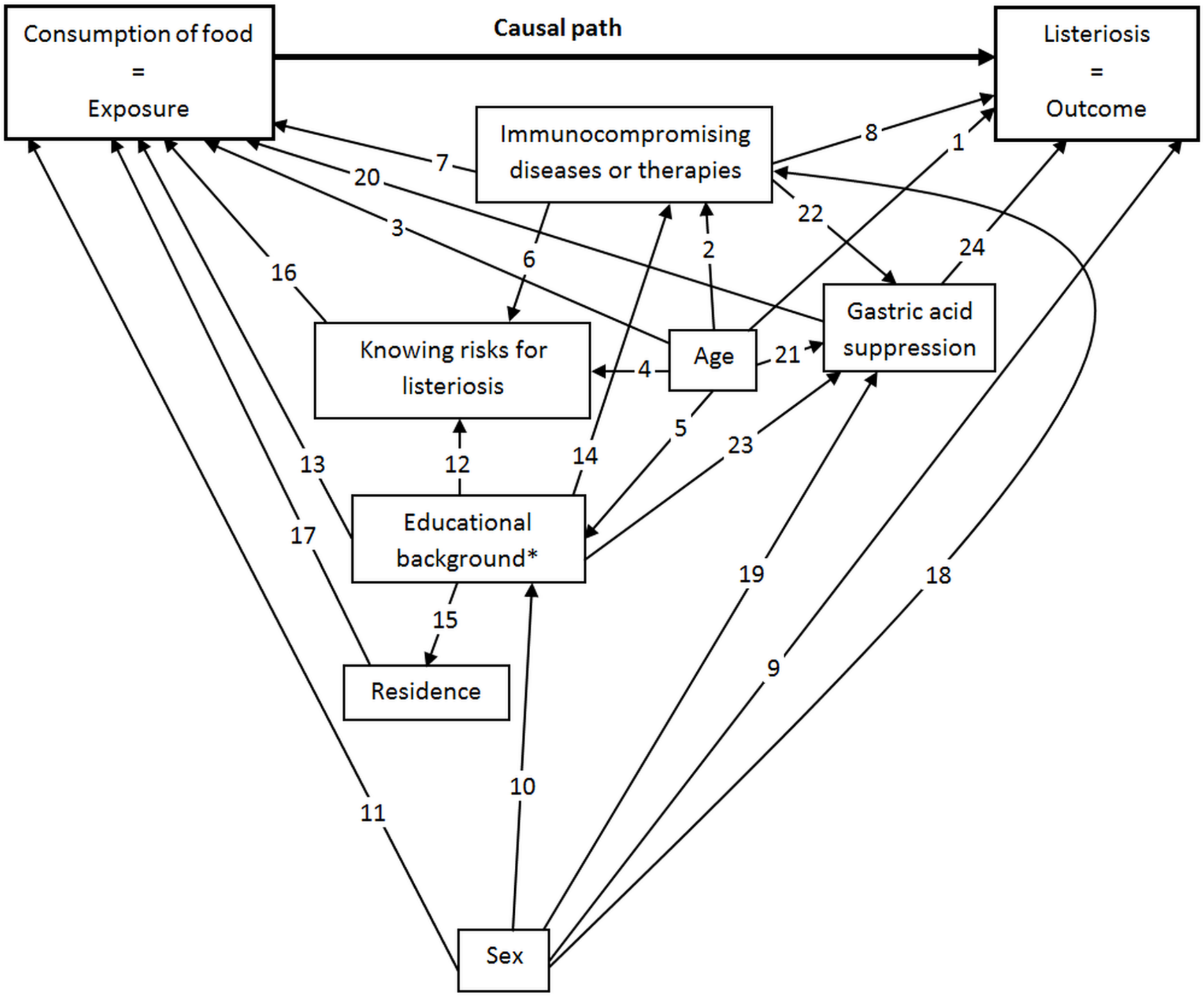

Fig 1. Hypothesized causal structure of exposure, host factors and non-pregnancy associated listeriosis, Germany, 2012–2013 [48–61].

doi:10.1371/journal.pone.0142986.g001

multivariable logistic regression model were investigated for statistical interactions using the likelihood-ratio test. Model specification was assessed with the link test.

Population attributable fractions for risk factors in the final multivariable model were estimated with 95% CI based on the method described by Greenland and Drescher [22, 23].

All statistical analyses were performed using Stata/IC 12.1 (StataCorp LP, USA).

Ethics Statement

Ethics approval was obtained from the Ethics committee of the Charité, Berlin (EA2/067/11) and the Federal Medical Association of Baden Württemberg (B-F-2012-038). Data collection and storage procedures were approved by the Federal Commissioner for Data Protection and Freedom of Information.

Results

We enrolled 109 case-patients out of 733 (15%) notified listeriosis patients (Fig 2) with a median age of 69 years (interquartile range, IQR 60–76); 60 (55%) were male. 103 (94%) case patients were hospitalized and four (4%) died. One-third (34%) of the case patients developed

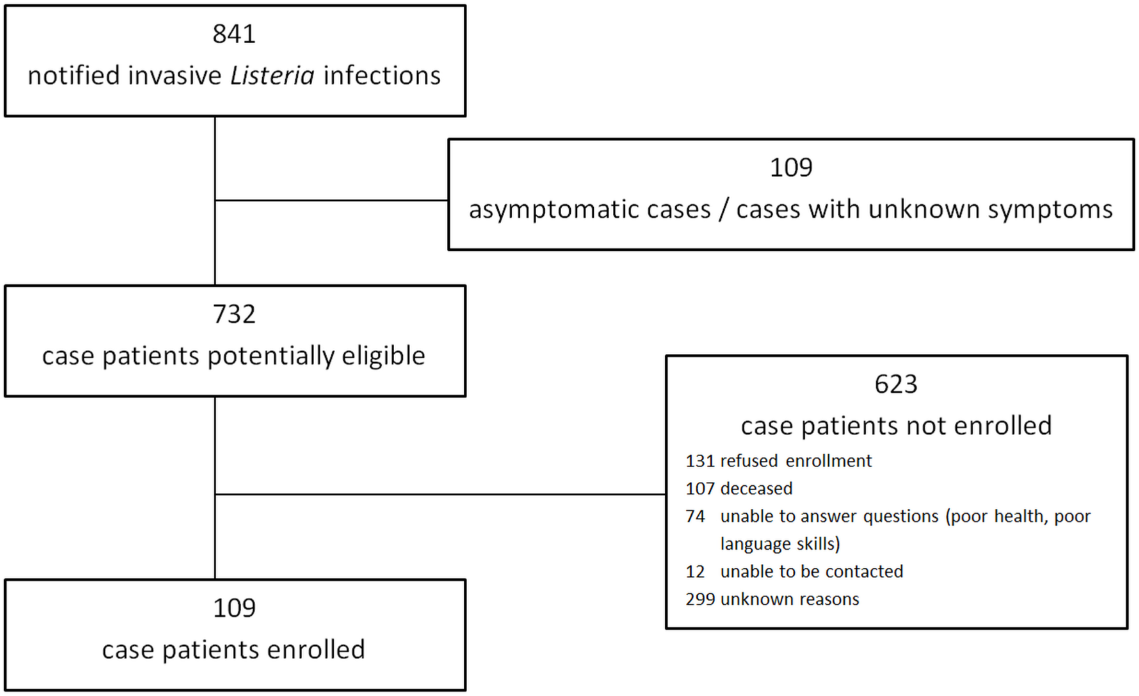

Fig 2. Enrollment of non-pregnancy associated listeriosis cases.

doi:10.1371/journal.pone.0142986.g002

meningitis (n = 37) and 20 (18%) developed sepsis. Sepsis was reported more frequently in cases older than 65 years compared to those who were younger (22% vs. 12%; χ^2 p = 0.17).

Cases included in the study were similar to notified cases not included with respect to sex, regional distribution of residence and proportion of sepsis. They were somewhat younger (median age: 69 years vs. 73 years; Wilcoxon rank-sum p = 0.02) and a higher proportion developed meningitis than those not included in the study (32% vs. 21%; χ^2 p = 0.01). Confirmed listeriosis related death was 2.5-times more frequently reported for cases not included in the study (10%; χ^2 p = 0.03).

75 (72%) case patients reported at least one of the underlying diseases or immunosuppressive therapies associated with listeriosis (Table 1). Immunosuppressive therapies in the 3 months prior to illness onset were mentioned by 46 (44%) case patients, particularly in cases aged $\leq$65 years (n = 23 [58%] vs. n = 23 [36%] in cases >65 years; χ^2 p = 0.03).

During the study period, no listeriosis outbreak with evidence for a food vehicle was identified in Germany. The National Reference Center for *Salmonella* and other bacterial enteric pathogens did not find evidence of outbreaks by subtyping isolates voluntarily submitted for outbreak surveillance Likewise, the statistical algorithms of the RKI did not flag a significant increase of listeriosis notifications during the time period.

Of 1990 recruited controls, eight had to be excluded due to their statement that they had suffered from listeriosis previously, resulting in 1982 control persons that were included in the study. Controls did not differ from cases with respect to age, sex and type of residence (Table 2), but had a higher proportion of high-level qualification than cases (33%; cases: 21%; χ^2 p = 0.01). A higher proportion of control persons knew about listeriosis (33%) than case patients prior to their illness (20%).

Knowledge about the disease was low in persons with immunocompromising diseases or who had received immunosuppressive therapy: only 13% of cases (7/54) and 10% of controls

Table 2. Demographic characteristics of the study population in a case control study of risk factors for non-pregnancy associated listeriosis in Germany, 2012–2013.

	Cases (n = 109)		Controls (n = 1982)		p-value*
	n	%	n	%	
Age					0.295
≤65	42/109	38.5	666/1982	33.6	
66–75	39/109	35.8	668/1982	33.7	
≥76	28/109	25.7	648/1982	32.7	
Sex					
Male	60/109	55.1	1101/1982	55.6	0.918
Educational background					0.021
Low	12/96	12.5	147/1918	7.7	
Medium	64/96	66.7	1137/1918	59.3	
High	20/96	20.8	634/1918	33.1	
Residence					0.620
Village	17/106	16.0	245/1824	13.4	
Town	53/106	50.0	992/1824	54.4	
Metropolis	36/106	34.0	587/1824	32.2	
Knowledge about listeriosis	20/101	19.8	655/1965	33.3	0.005
Immunocompromising diseases or therapies[a]					<0.001
No	29/104	27.9	1294/1890	68.5	
Immunocompromising disease[b]	29/104	27.9	369/1890	19.5	
Immunosuppressive therapy[c]	46/104	44.2	227/1890	12.0	

[a] mutual exclusive categories

[b] diabetes, renal disease, liver disease, autoimmune disorder, hematological cancer (within last 5 years), solid organ transplantation and/or splenectomy

[c] chemotherapy, radiation therapy, hemodialysis and/or immunosuppressive medication (all within the last 3 months)

* χ^2 test

doi:10.1371/journal.pone.0142986.t002

(58/589; χ^2 p = 0.47) knew transmission risks of listeriosis. Transmission via food was least known among persons under radiation therapy in the preceeding 3 months (1/21; 5%).

In univariable analysis, several underlying conditions were positively associated with listeriosis, including diabetes, renal disease or chemotherapy within the last 3 months as well as gastric acid suppression (Table 1). Furthermore, the following food-related exposures were significantly associated with increased odds of listeriosis ($p<0.05$): consumption of cold cooked sausages, pre-sliced cheese, packaged cheese, raw fermented spreadable sausages, cheese purchased in a supermarket or discounter, foods consumed in a hospital or nursing home, and foods consumed in a restaurant or canteen at least once a weak. We found 23 exposures negatively associated with listeriosis (Table 3).

Based on the causal diagram (Fig 1), we identified age, sex, immunocompromising disease or immunosuppressive therapy, and gastric acid suppression as a minimally sufficient adjustment set, which was forced into the multivariable model (Table 4). Immunosuppressive therapies and immunocompromising diseases remained strongly associated with listeriosis in the multivariable model. The odds of listeriosis in persons who had received immunosuppressive therapy in the last 3 months was approximately 9 times that of persons without any immunocompromising conditions (OR 8.75, 95% CI 4.91–15.58) and almost 3 times that of persons with immunocompromising diseases but without immunosuppressive therapy (OR 2.74, 95% CI 1.44–5.20). Furthermore, gastric acid suppression raised the odds for listeriosis by factor 3

Table 3. Food exposures statistically significantly associated with non-pregnancy associated listeriosis, Germany, 2012–2013: age-adjusted univariable logistic regression analysis.

Consumption of . . .*	Cases exposed		Controls exposed				
	n	%	n	%	OR	95% CI	p-value
Cold cooked sausages (e.g., frankfurters, wieners, bockwurst), not reheated	44/101	43.6	455/1976	23.0	2.54	1.69–3.81	<0.001
Pre-sliced cheese (in contrast to uncut cheese)	48/100	48.0	533/1948	28.4	2.34	1.56–3.51	<0.001
Packaged cheese (in contrast to unpackaged cheese)	59/100	59.0	803/1967	40.8	2.11	1.40–3.18	<0.001
Raw fermented spreadable sausages (e.g. Teewurst, Zwiebelmettwurst)	60/103	58.3	958/1975	48.5	1.51	1.01–2.26	0.043
Raw milk	2/106	1.9	254/1971	12.9	0.13	0.03–0.55	0.005
Ewe's cheese, goat cheese	16/101	15.8	956/1974	48.4	0.18	0.11–0.31	<0.001
Raw milk cheese	20/54	37.0	1034/1417	73.0	0.21	0.12–0.37	<0.001
Cold seafood (e.g., prawns, shrimps)	11/102	10.8	538/1980	27.2	0.31	0.17–0.59	<0.001
Mozzarella, feta cheese etc.	35/104	33.7	1101/1975	55.8	0.34	0.22–0.72	<0.001
Fresh cheese (e.g., cream cheese, cottage cheese, mascarpone)	43/102	42.2	1314/1962	67.0	0.35	0.23–0.52	<0.001
Carved and packaged raw fruit, fruit salads etc.	8/105	7.6	366/1978	18.5	0.37	0.18–0.78	0.008
Pasteurized milk	53/103	51.5	1451/1981	73.3	0.38	0.25–0.57	<0.001
Blue-veined cheese (e.g., roquefort, gorgonzola)	24/101	23.8	891/1976	45.1	0.38	0.24–0.61	<0.001
Acid curd cheese	23/99	23.2	808/1973	41.0	0.45	0.28–0.72	0.001
Red-smear cheese (e.g., Limburger, Romadur, Munster cheese)	23/100	23.0	745/1968	38.0	0.49	0.31–0.80	0.004
Semi-soft cheese (e.g., butter cheese, Esrom, Havarti)	33/98	33.7	978/1967	49.7	0.50	0.33–0.77	0.002
Cooked, roast or barbecued meat	86/103	83.5	1771/1980	89.4	0.55	0.32–0.95	0.034
White mould cheese (e.g., camembert, brie)	60/101	59.4	1420/1976	71.9	0.57	0.38–0.86	0.007
Dry-cured ham (e.g., Serrano ham, Parma ham)	70/104	67.3	1547/1979	78.2	0.57	0.37–0.86	0.008
Deli salads	41/103	39.8	1027/1977	52.0	0.59	0.40–0.89	0.012
Any cheese with negative association to listeriosis	92/103	89.3	1859/1966	94.6	0.48	0.25–0.94	0.031
Cheese from supermarket or discounter	100/105	95.2	1737/1971	88.1	2.66	1.07–6.60	0.035
Cheese from specialized shop	8/105	7.6	630/1959	32.2	0.17	0.08–0.36	<0.001
Meat/sausage from farmer's market, direct sale	6/104	5.8	481/1968	24.4	0.19	0.08–0.43	<0.001
Fish/seafood from travelling hawker, mail-order selling	2/103	1.9	163/1965	8.3	0.23	0.06–0.93	0.039
Fish/seafood from specialized shop	5/98	5.1	294/1654	17.8	0.25	0.10–0.62	0.003
Cheese from farmer's market, direct sale	6/104	5.8	369/1981	18.6	0.26	0.11–0.59	0.001
Fish/seafood from farmer's market, direct sale	9/101	8.9	374/1968	19.0	0.42	0.21–0.83	0.013
Foods supplied in a hospital, rest home, nursing home	11/108	10.2	78/1981	3.9	2.86	1.47–5.57	0.002
Foods purchased by other family or household members	85/108	78.7	1216/1980	61.4	2.27	1.41–3.63	<0.001
Foods supplied in a restaurant, canteen etc. at least once a week	32/104	30.8	423/1981	21.4	1.57	1.01–2.44	0.023
Foods from own purchase	86/109	78.9	1762/1982	88.9	0.43	0.26–0.70	0.002

* within the last 4 weeks prior to onset of illness (cases) and prior to the interview (controls), respectively

Exposures not statistically significantly associated with N-PA listeriosis: Consumption of packaged meat and sausage products, salami, scalded deli meat, pâté, sliced cooked deli meat, sliced aspics, sliced blood sausage, raw minced meat, packaged fish/seafood, raw fish (sushi), smoked or graved fish, marinated fish, medium-hard/hard cheese, carved and packaged raw vegetables, meat/sausages from supermarket or discounter, butcher shop, wholefood shop, filling station/station kiosk, travelling hawker or mail-order selling, self-made meat/sausages, fish/seafood from supermarket or discounter, wholefood shop, self-made fish/seafood, cheese from wholefood shop, filling station/station kiosk, travelling hawker or mail-order selling, self-made cheese, and food supplied by delivery service (pizza service, meals on wheels etc.) or by assisted living.

doi:10.1371/journal.pone.0142986.t003

(OR 2.96, 95% CI 1.40–6.25). The consumption of cold cooked sausages and preferred consumption of packaged cheese and sliced cheese were significantly associated with N-PA listeriosis.

Table 4. Risk factors for sporadic non-pregnancy associated listeriosis in Germany, 2012–2013: multivariable logistic regression analysis.

	OR	95% CI	p-value	PAF (%)	95% CI (%)
Immunocompromising diseases or therapies*[a]					
immunosuppressive therapy within the last 3 months	8.75	4.91–15.58	<0.001		
immunocompromising disease without immunosuppressive therapy	2.73	1.44–5.20	0.002		
none	Ref.				
Gastric acid suppression*	2.96	1.40–6.25	0.005	9.3	5.7–12.8
Consumption of (not-reheated) cold cooked sausages	2.60	1.56–4.35	<0.001	24.5	16.3–31.9
Preferred consumption of packaged cheese	2.09	1.25–3.49	0.005	30.7	14.7–43.8
Preferred consumption of pre-sliced cheese	2.19	1.31–3.66	0.003	27.2	14.4–38.0
Consumption of any cheese with negative association to listeriosis[b]	0.33	0.15–0.73	0.007		
Consumption of pasteurized milk	0.47	0.28–0.77	0.003		
Consumption of carved and packaged raw fruit, fruit salads etc.	0.27	0.11–0.72	0.008		
Consumption of deli salads	0.46	0.27–0.78	0.004		
Age*					
≤65	Ref.				
66–75	0.63	0.35–1.12	0.116		
≥76	0.54	0.29–1.01	0.056		
Female sex*	0.90	0.54–1.48	0.670		

[a] see Table 1

[b] raw milk cheese; ewe's cheese, goat cheese; mozzarella, feta cheese; fresh cheese; blue-veined cheese; acid curd cheese; red-smear cheese; semi-soft cheese; white-mould cheese

* were identified as the minimally sufficient adjustment set in a directed acyclic graph and thus included in the multivariable model

doi:10.1371/journal.pone.0142986.t004

The model specification was confirmed by the link test. We did not find significant interactions between the model variables.

The population attributable fraction (PAF) of the food-variables, i.e. consumption of non-reheated cold cooked sausages, preferably packaged cheese or preferably sliced cheese was approximately 25% each (Table 4). The total PAF for all identified risk foods was 58.9% (95% CI 49.8%–66.4%).

Discussion

This population-based case-control study of risk factors for sporadic N-PA listeriosis in Germany yielded three main findings. First, reduced immunocompetency is the major risk factor for listeriosis. Second, foods associated with listeriosis, i.e. cold cooked sausages and both packaged cheese and pre-sliced cheese, are ready-to-eat, usually have a long shelf-life, and were popular among the general adult population in Germany. Together, they accounted for 59% of the cases in Germany. Third, about 80% of cases had never heard of listeriosis prior to their illness and only 6% of the immunocompromised control population knew about the principally food-borne nature of the disease, indicating an as yet under-utilized potential for risk communication and disease prevention.

Our study confirmed [24–26] and quantified the outstanding role of reduced immunocompetency for development of N-PA listeriosis. 44% of cases had received an immunosuppressive therapy within the 3 months prior to illness onset and another 28% had an underlying immunocompromising disease, such as diabetes. The disease risk appeared to correlate with degree of reduced immunocompetency (assuming that patients who recently received immunosuppressive therapy were, on average, more immunocompromised than those with "only"

immunocompromising disease(s)). Moreover, knowledge about listeriosis transmission pathways was low among immunocompromised persons and appeared to be particularly lacking among persons who had received chemotherapy or radiation therapy within the last 3 months. To our knowledge, dietary recommendations for prevention of food-borne infections do not exist for this subgroup of patients in Germany. Hence, healthcare institutions in Germany should be aware of the increased risk of immunocompromised persons for listeriosis if catering food for this population.

Gastric acid suppression was associated with listeriosis in former studies [7–9, 26] but failed to be an independent risk factor when controlled for underlying immunocompromising conditions [6, 27]. In our study, gastric acid suppression remained associated with listeriosis when adjusted for immunocompromising diseases and immunosuppressive therapy. Yet, all cases with gastric acid suppression and complete information for underlying conditions (n = 16) suffered also from at least one underlying immunocompromising disease (n = 7) or had received immunosuppressive therapy (n = 9). Thus, use of these drugs warrants particular care in already immunocompromised persons.

The identified ready-to-eat foods in this study, which are all frequently consumed in Germany, have not yet been considered risky foods in Germany, but they are plausible vehicles for sporadic N-PA listeriosis. Cold cooked sausages are heated during manufacturing but they are susceptible to post-production contamination until package [28]. They also support relatively rapid growth of *L. monocytogenes* under refrigerated storage conditions [29, 30] and *L. monoctyogenes* is frequently detected in these products [31]. Furthermore, our finding of a significant association between listeriosis and consumption of cold cooked sausages that were not reheated before eating are consistent with a quantitative risk assessment of foodborne *L. monocytogenes* among ready-to-eat foods [32] in which non reheated frankfurters (a cold cooked sausage), were classified as a (very) high risk food.

Many different types of cheese–made of unpasteurized milk as well as from pasteurized milk–were identified as vehicle in listeriosis outbreaks [33] or have been the subject of an alert within the European Commission-operated Rapid Alert System for Food and Feed because of contamination with *L. monocytogenes* [34]. In keeping with this, we identified no single type of cheese but two retail forms of cheese, irrespective of the cheese type, that were statistically significantly associated with listeriosis. "Packaged cheese"combined various types of soft, semi-hard or hard cheese sold as whole pieces as well as pre-sliced. Notably, packaged cheese for self-service normally has an extended shelf-life and supports the growth of *L. monocytogenes* [35] after possible post-production contamination [36]. In contrast, cheese bought at counters is frequently cross-contaminated with mold and therefore has a shorter durability [37]. Hard cheese is the top-selling type of cheese in Germany and is often consumer-friendly offered as pre-sliced product, particularly in self-service retail, which accounts for 93% of cheese sales [38]. Slicing equipment is a known source for *Listeria* contamination in the food production and processing environment [39, 40]. Low level contamination of sliced cheese products with *L. monocytogenes* is not unusual [41] and sometimes even sufficient to cause outbreaks [42]. In our study, 18% of the control population reported daily consumption of sliced cheeses, a further 54% at least once per week. The popularity of pre-sliced and hard cheese in the general population may outweigh the apparently low prevalence of *L. monocytogenes* in hard cheese (0.4%), seldom in high concentrations (approx. 14% of positive samples), in a recent food survey in Germany [43].

The ability of *L. monocytogenes* to grow at low temperatures is well known [44]. Therefore, the European Commission Regulation on microbiological criteria for foodstuffs (EC No 2073/2005) requires that the concentration of *L. monoytogenes* in ready-to-eat foods, such as cold cooked sausages or pre-sliced and packaged cheeses, does not exceed 100 colony forming units

per gram throughout the shelf-life. Our study results indicate (i) that this safety level may be insufficient to protect immunocompromised persons from acquiring listeriosis or (ii) that these ready-to-eat foods were consumed after the 'use by' date or the minimum durability date, thereby allowing *L. monocytogenes* to multiply to concentrations above the safety level, or both. That the retail form (e.g. pre-sliced/packaged) rather than a specific cheese type appeared to be important in this study, coupled with the observation that *L. monocytogenes* is infrequently present in these cheeses, lends credibility to the hypothesis that the time-interval between production and consumption of these cheeses, and possibly also of cold cooked sausages, plays a pivotal role for acquiring N-PA listeriosis in Germany. By eliminating the risk associated with any one of these foods, 25% of cases in Germany could be prevented (and 59% by eliminating the risk of all three) indicating their relevance for listeriosis in Germany and the large disease prevention potential.

Similar to other case-control studies of risk factors for sporadic listeriosis [6, 9], our study did not find a statistical association of listeriosis with many typical high risk foods, such as ready-to-eat fish products (graved/smoked fish) or certain types of unpasteurized cheese. On the contrary, some typically risky foods (e.g. raw milk, red smear cheese, cold seafood) were negatively associated with listeriosis. We offer the following explanations for this finding. Firstly, many of the high-risk foods have become prominent through outbreaks (in other countries) and it is unclear whether outbreak-related risk factors equally apply to sporadic cases (in Germany). Secondly, some high-risk foods (e.g. graved/smoked fish) are characterized by a high prevalence of *L. monocytogenes*. However, the overall prevalence of *L. monocytogenes* might not correlate well with the prevalence of pathogenic *L. monocytogenes*. Frequent occurrence of apathogenic or low virulent strains in retail foods and processing environment has been described [45, 46]. Furthermore, other factors such as food-processing before consumption, the food matrix's ability to support bacterial growth, and the time between production and consumption of the food may confound the association of a specific food-item with listeriosis.

Our study is subject to potential biases. We enrolled a smaller number of cases than planned which reduced the ability of the study to identify foods associated with listeriosis. Furthermore, cases in this study were somewhat younger and tended to have milder diseases than cases not included in the study and food consumption might depend on age. We aimed to include an age-adjusted representative sample of the German adult population as the reference population in this study, but our controls, as is common for telephone surveys [47], disproportionately contained persons with a high socioeconomic status (SES) (when approximated by educational background). Compared to the German microcensus of 2012, persons with highest educational background were 2-fold over-represented in our control population (33% vs. 16% at microcensus). As consumption of various food items, especially cheese types, was positively correlated with educational background in our large control population (data not shown), these foods were found to be rather negatively than positively correlated with N-PA listeriosis. Although we controlled for the confounding impact of SES analytically in the multivariable model, we cannot exclude that other factors, uncontrolled in this study, relate to consumption patterns through means other than SES.

We did neither enquire about specific types of cold cooked sausages nor about their package and shelf life. Thus, further studies are needed to better characterize the risk associated with this particular food item.

Conclusions

Our study confirmed the outstanding role of reduced immunocompetency for acquiring N-PA listeriosis, and identified ready-to-eat foods as risk factors that are frequently consumed in Germany. These foods usually have an extended shelf-life, are biologically plausible, but have not yet been considered high-risk foods for listeriosis in Germany. To reduce their risk, immunocompromised persons, particularly those receiving immunosuppressive therapy, should consume packaged and pre-sliced cheese (and possibly cold cooked sausages) well before their 'use-by' date. It is possible that the microbiological criteria for *L. monocytogenes* in ready-to-eat foods are inadequate to protect persons with markedly reduced immunocompetency from acquiring listeriosis. Specific recommendations for this population should be formulated or amended and heed the findings of this study.

Acknowledgments

We thank all study participants and the local and state health authorities for their support. We are grateful to many colleagues in the RKI CATI Call centre, who performed the control interviews, Hannes Ulrich, Constanze Knebel and Elise Curo Gutierrez for study assistance and data entry. We thank Kevin Pollock for language editing of the manuscript.

Author Contributions

Conceived and designed the experiments: AMB KS DW. Performed the experiments: KP. Analyzed the data: KP. Wrote the paper: KP DW. Designed and performed the population-based telephone survey: PS MW. Assisted with epidemiological interpretation: KS DW. Critically revised the manuscript: AMB PS MW KS.

References

1. Bavishi C, Dupont HL. Systematic review: the use of proton pump inhibitors and increased susceptibility to enteric infection. Aliment Pharmacol Ther. 2011; 34:1269–81. doi: 10.1111/j.1365-2036.2011.04874.x PMID: 21999643
2. Mead PS, Slutsker L, Dietz V, McCaig LF, Bresee JS, Shapiro C, et al. Food-related illness and death in the United States. Emerg Infect Dis. 1999; 5:607–25. PMID: 10511517
3. Cartwright EJ, Jackson KA, Johnson SD, Graves LM, Silk BJ, Mahon BE. Listeriosis outbreaks and associated food vehicles, United States, 1998–2008. Emerg Infect Dis. 2013; 19:1–9. doi: 10.3201/eid1901.120393 PMID: 23260661
4. Centers for Disease Control and Prevention. Vital signs: Listeria illnesses, deaths, and outbreaks—United States, 2009–2011. MMWR Morb Mortal Wkly Rep. 2013; 62:448–52. PMID: 23739339
5. Miettinen MK, Siitonen A, Heiskanen P, Haajanen H, Bjorkroth KJ, Korkeala HJ. Molecular epidemiology of an outbreak of febrile gastroenteritis caused by Listeria monocytogenes in cold-smoked rainbow trout. J Clin Microbiol. 1999; 37:2358–60. PMID: 10364616
6. Friesema IH, Kuiling S, van der Ende A, Heck ME, Spanjaard L, van Pelt W. Risk factors for sporadic listeriosis in the Netherlands, 2008 to 2013. Euro Surveill. 2015; 20(31). PMID: 26290429
7. Dalton CB, Merritt TD, Unicomb LE, Kirk MD, Stafford RJ, Lalor K, et al. A national case-control study of risk factors for listeriosis in Australia. Epidemiol Infect. 2011; 139:437–45. doi: 10.1017/S0950268810000944 PMID: 20429970
8. Jensen A, Frederiksen W, Gerner-Smidt P. Risk factors for listeriosis in Denmark, 1989–1990. Scand J Infect Dis. 1994; 26:171–8. PMID: 8036473
9. Varma JK, Samuel MC, Marcus R, Hoekstra RM, Medus C, Segler S, et al. Listeria monocytogenes Infection from Foods prepared in a Commercial Establishment: A Case-Control Study of Potential Sources of Sporadic Illness in the United States. Clin Infect Dis. 2007; 44:521–8. PMID: 17243054
10. Gillespie IA, Mook P, Little CL, Grant K, Adak GK. Listeria monocytogenes infection in the over-60s in England between 2005 and 2008: A retrospective case-control study utilizing market research panel data. Foodborne Pathog Dis. 2010; 7:1373–9. doi: 10.1089/fpd.2010.0568 PMID: 20586610

11. Werber D, Hille K, Frank C, Dehnert M, Altmann D, Muller-Nordhorn J, et al. Years of potential life lost for six major enteric pathogens, Germany, 2004–2008. Epidemiol Infect. 2013; 141:961–8. doi: 10.1017/S0950268812001550 PMID: 22813426

12. Häder S, Gabler S. [A new sampling design for telephone surveys in Germany.] In: Gabler S, Häder S, Hoffmeyer-Zlotnik JHP, editors. Telefonstichproben in Deutschland. Opladen, Wiesbaden: Westdeutscher Verlag; 1998. p. 69–88.

13. Lampert T, Kroll LE, Muters S, Stolzenberg H. [Measurement of the socioeconomic status within the German Health Update 2009 (GEDA)]. Bundesgesundheitsblatt Gesundheitsforschung Gesundheitsschutz. 2013; 56:131–43. doi: 10.1007/s00103-012-1583-3 PMID: 23275948

14. Bohnsack JF, Brown EJ. The role of the spleen in resistance to infection. Annu Rev Med. 1986; 37:49–59. PMID: 3518612

15. de Marie S. Diseases and drug-related interventions affecting host defence. Eur J Clin Microbiol Infect Dis. 1993; 12 Suppl 1:S36–41. PMID: 8477761

16. Gan YH. Host susceptibility factors to bacterial infections in type 2 diabetes. PLoS Pathog. 2013; 9: e1003794. doi: 10.1371/journal.ppat.1003794 PMID: 24385898

17. Koscielska-Kasprzak K, Bartoszek D, Myszka M, Zabinska M, Klinger M. The complement cascade and renal disease. Arch Immunol Ther Exp (Warsz). 2014; 62:47–57.

18. Sheth K, Bankey P. The liver as an immune organ. Curr Opin Crit Care. 2001; 7:99–104. PMID: 11373518

19. Greenland S, Pearl J, Robins JM. Causal diagrams for epidemiologic research. Epidemiology. 1999; 10:37–48. PMID: 9888278

20. Textor J, Hardt J, Knuppel S. DAGitty: a graphical tool for analyzing causal diagrams. Epidemiology. 2011; 22:745. doi: 10.1097/EDE.0b013e318225c2be PMID: 21811114

21. Peduzzi P, Concato J, Kemper E, Holford TR, Feinstein AR. A simulation study of the number of events per variable in logistic regression analysis. J Clin Epidemiol. 1996; 49:1373–9. PMID: 8970487

22. Greenland S, Drescher K. Maximum likelihood estimation of the attributable fraction from logistic models. Biometrics. 1993; 49:865–72. PMID: 8241375

23. Newson RB. Attributable and unattributable risks and fractions and other scenario comparisons. The Stata Journal. 2013; 13:672.

24. Goulet V, Hebert M, Hedberg C, Laurent E, Vaillant V, De Valk H, et al. Incidence of listeriosis and related mortality among groups at risk of acquiring listeriosis. Clin Infect Dis. 2012; 54:652–60. doi: 10.1093/cid/cir902 PMID: 22157172

25. Mook P, Jenkins JM, O'Brien SJ, Gillespie IA. Existing medications among non-pregnancy-related listeriosis patients in England, 2007–2009. Epidemiol Infect. 2013; 141:36–44. doi: 10.1017/S095026881200026X PMID: 22404843

26. Mook P, O'Brien SJ, Gillespie IA. Concurrent conditions and human listeriosis, England, 1999–2009. Emerg Infect Dis. 2011; 17:38–43. doi: 10.3201/eid1701.101174 PMID: 21192852

27. Goulet V, Marchetti P. Listeriosis in 225 non-pregnant patients in 1992: clinical aspects and outcome in relation to predisposing conditions. Scand J Infect Dis. 1996; 28:367–74. PMID: 8893400

28. Wenger JD, Swaminathan B, Hayes PS, Green SS, Pratt M, Pinner RW, et al. Listeria monocytogenes contamination of turkey franks: Evaluation of a production facility. J Food Prot. 1990; 53:1015–9.

29. Glass KA, Doyle MP. Fate of Listeria monocytogenes in processed meat products during refrigerated storage. Appl Environ Microbiol. 1989; 55:1565–9. PMID: 2504110

30. Pal A, Labuza TP, Diez-Gonzalez F. Safety-based shelf life model for frankfurters based on time to detect Listeria monocytogenes with initial inoculum below detection limit. J Food Prot. 2009; 72:1878–84. PMID: 19777889

31. Wallace FM, Call JE, Porto AC, Cocoma GJ, Luchansky JB. Recovery rate of Listeria monocytogenes from commercially prepared frankfurters during extended refrigerated storage. J Food Prot. 2003; 66:584–91. PMID: 12696680

32. FDA/FSIS. Quantitative Assessment of Relative Risk to Public Health from Foodborne Listeria monocytogenes Among Selected Categories of Ready-to-Eat Foods. 2003. Food and Drug Administration, United States Department of Agriculture, Centers for Disease Control and Prevention.

33. Gould LH, Mungai E, Behravesh CB. Outbreaks attributed to cheese: differences between outbreaks caused by unpasteurized and pasteurized dairy products, United States, 1998–2011. Foodborne Pathog Dis. 2014; 11:545–51. doi: 10.1089/fpd.2013.1650 PMID: 24750119

34. Koch J, Dworak R, Prager R, Becker B, Brockmann S, Wicke A, et al. Large Listeriosis Outbreak Linked to Cheese Made from Pasteurized Milk, Germany, 2006–2007. Foodborne Pathog Dis. 2010; 7:1581–4. doi: 10.1089/fpd.2010.0631 PMID: 20807110

35. Whitley E, Muir D, Waites WM. The growth of Listeria monocytogenes in cheese packed under a modified atmosphere. J Appl Microbiol. 2000; 88:52–7. PMID: 10735243

36. Kousta M, Mataragas M, Skandamis P, Drosinos EH. Prevalence and sources of cheese contamination with pathogens at farm and processing levels. Food Control. 2010; 21:805–15.

37. Bavarian Consumer Organisation. [Market check-up—Shelf-life of pre-sliced cheese at self-service counters.] 2013. Available: http://www.verbraucherzentrale-bayern.de/mediabig/224717A.pdf. Accessed 13 Jan 2015.

38. Anonymus. Ist die Käsetheke "uncool"? Milch-Marketing. 2013:16–8.

39. Johansson T, Rantala L, Palmu L, Honkanen-Buzalski T. Occurrence and typing of Listeria monocytogenes strains in retail vacuum-packed fish products and in a production plant. Int J Food Microbiol. 1999; 47:111–9. PMID: 10357279

40. Sheen S, Hwang CA. Modeling transfer of Listeria monocytogenes from slicer to deli meat during mechanical slicing. Foodborne Pathog Dis. 2008; 5:135–46. doi: 10.1089/fpd.2007.0049 PMID: 18361687

41. Little CL, Rhoades JR, Sagoo SK, Harris J, Greenwood M, Mithani V, et al. Microbiological quality of retail cheeses made from raw, thermized or pasteurized milk in the UK. Food Microbiol. 2008; 25:304–12. doi: 10.1016/j.fm.2007.10.007 PMID: 18206773

42. Yde M, Naranjo M, Mattheus W, Stragier P, Pochet B, Beulens K, et al. Usefulness of the European Epidemic Intelligence Information System in the management of an outbreak of listeriosis, Belgium, 2011. Euro Surveill. 2012; 17(38).

43. Hartung M, Tenhagen BA, Käsbohrer A. [Zoonotic pathogens in Germany, 2012] Berlin: Federal Institute for Risk Assessment (BfR), 2014. Available: http://www.bfr.bund.de/cm/350/erreger-von-zoonosen-in-deutschland.pdf. Accessed 6 May 2015.

44. Walker SJ, Archer P, Banks JG. Growth of Listeria monocytogenes at refrigeration temperatures. J Appl Bacteriol. 1990; 68:157–62. PMID: 2108109

45. Norton DM, Scarlett JM, Horton K, Sue D, Thimothe J, Boor KJ, et al. Characterization and pathogenic potential of Listeria monocytogenes isolates from the smoked fish industry. Appl Environ Microbiol. 2001; 67:646–53. PMID: 11157227

46. Roche SM, Gracieux P, Albert I, Gouali M, Jacquet C, Martin PM, et al. Experimental validation of low virulence in field strains of Listeria monocytogenes. Infect Immun. 2003; 71:3429–36. PMID: 12761127

47. Voigt LF, Koepsell TD, Daling JR. Characteristics of telephone survey respondents according to willingness to participate. Am J Epidemiol. 2003; 157:66–73. PMID: 12505892

48. Gomez CR, Boehmer ED, Kovacs EJ. The aging innate immune system. Curr Opin Immunol. 2005; 17:457–62. PMID: 16084711

49. Pouillot R, Hoelzer K, Jackson KA, Henao OL, Silk BJ. Relative risk of listeriosis in Foodborne Diseases Active Surveillance Network (FoodNet) sites according to age, pregnancy, and ethnicity. Clin Infect Dis. 2012; 54 Suppl 5:S405–10. doi: 10.1093/cid/cis269 PMID: 22572661

50. Fuchs J, Busch M, Lange C, Scheidt-Nave C. Prevalence and patterns of morbidity among adults in Germany. Results of the German telephone health interview survey German Health Update (GEDA) 2009. Bundesgesundheitsblatt Gesundheitsforschung Gesundheitsschutz. 2012; 55:576–86. doi: 10.1007/s00103-012-1464-9 PMID: 22441528

51. Max Rubner Institute (Ed.). [National Food Consumption Survey II. Report on findings—Part 2], 2008. Available: www.was-esse-ich.de/uploads/media/NVSII_Abschlussbericht_Teil_2.pdf. Accessed 13 Jan 2015.

52. Jungjohann SM, Luhrmann PM, Bender R, Blettner M, Neuhauser-Berthold M. Eight-year trends in food, energy and macronutrient intake in a sample of elderly German subjects. Br J Nutr. 2005; 93:361–78. PMID: 15877876

53. Yeretssian G, Doiron K, Shao W, Leavitt BR, Hayden MR, Nicholson DW, et al. Gender differences in expression of the human caspase-12 long variant determines susceptibility to Listeria monocytogenes infection. Proc Natl Acad Sci U S A. 2009; 106:9016–20. doi: 10.1073/pnas.0813362106 PMID: 19447924

54. Hulshof KF, Brussaard JH, Kruizinga AG, Telman J, Lowik MR. Socio-economic status, dietary intake and 10 y trends: the Dutch National Food Consumption Survey. Eur J Clin Nutr. 2003; 57:128–37. PMID: 12548307

55. Lampert T, Kroll LE, von der Lippe E, Muters S, Stolzenberg H. [Socioeconomic status and health: results of the German Health Interview and Examination Survey for Adults (DEGS1)]. Bundesgesundheitsblatt Gesundheitsforschung Gesundheitsschutz. 2013; 56:814–21. doi: 10.1007/s00103-013-1695-4 PMID: 23703503

56. Hoffmann F, Glaeske G, Schmiemann G. [Increased prescribing of proton pump inhibitors in ambulatory care over the years 2005–2013]. Z Gastroenterol. 2015; 53:95–100. doi: 10.1055/s-0034-1384973 PMID: 25251751

57. Nocon M, Keil T, Willich SN. Prevalence and sociodemographics of reflux symptoms in Germany—results from a national survey. Aliment Pharmacol Ther. 2006; 23:1601–5. PMID: 16696809

58. van Boxel OS, Hagenaars MP, Smout AJ, Siersema PD. Socio-demographic factors influence chronic proton pump inhibitor use by a large population in the Netherlands. Aliment Pharmacol Ther. 2009; 29:571–9. doi: 10.1111/j.1365-2036.2008.03900.x PMID: 19035978

59. Schlech WF 3rd, Chase DP, Badley A. A model of food-borne Listeria monocytogenes infection in the Sprague-Dawley rat using gastric inoculation: development and effect of gastric acidity on infective dose. Int J Food Microbiol. 1993; 18:15–24. PMID: 8466809

60. Ho JL, Shands KN, Friedland G, Eckind P, Fraser DW. An outbreak of type 4b Listeria monocytogenes infection involving patients from eight Boston hospitals. Arch Intern Med. 1986; 146:520–4. PMID: 3954524

61. Ahrens D, Behrens G, Himmel W, Kochen MM, Chenot JF. Appropriateness of proton pump inhibitor recommendations at hospital discharge and continuation in primary care. Int J Clin Pract. 2012; 66:767–73. doi: 10.1111/j.1742-1241.2012.02973.x PMID: 22805269
